# Multiple mechanisms enable broad-spectrum activity of the *Pelargonium sidoides* root extract EPs 7630 against acute respiratory tract infections

**DOI:** 10.3389/fphar.2024.1455870

**Published:** 2024-10-14

**Authors:** Jindrich Cinatl, Mark N. Wass, Martin Michaelis

**Affiliations:** ^1^ Interdisciplinary Laboratory for Tumour and Virus Research, Dr Petra Joh Research Institute, Frankfurt am Main, Germany; ^2^ School of Biosciences, University of Kent, Canterbury, United Kingdom

**Keywords:** herbal drugs, antiviral, antibacterial, interferon, immunomodulation, infectious disease, virus, bacteria

## Abstract

There is clinical evidence showing that the *Pelargonium sidoides* root extract EPs 7630 is a safe and effective treatment for a range of acute infectious respiratory illnesses. Moreover, EPs 7630 has been shown to reduce the use of antibiotics, which is important in the context of rising antibiotic resistance levels. A wide range of mechanisms appears to contribute to the beneficial effects of EPs 7630, e.g. antibacterial, antiviral, immunomodulatory, and epithelial barrier effects. This broad spectrum of pharmacological activities seems to enable the clinical activity of EPs 7630 against multiple respiratory infections. In particular, the combination of antiviral and immunomodulatory effects may enable EPs 7630 to tackle acute viral respiratory infections both in early stages of the disease process, which are driven by virus replication, as well as in later stages, which are caused by an overshooting immune response. Hence, EPs 7630 is a prime example of a plant extract with evidence-based clinical efficacy, including a solid understanding of the underlying mechanisms of action. The example of EPs 7630 demonstrates that plant extracts have a potential role as evidence-based clinical treatments and that they deserve pre-clinical and clinical testing and investigation in the same way as any other drug class.

## 1 Introduction

The roots and rhizomes of *Pelargonium sidoides* DC. (Geraniaceae, known as African geranium or South African geranium) have been used for centuries as herbal remedies as part of traditional medicine practices in South Africa for gastrointestinal disorders and respiratory conditions including tuberculosis (Bladt and Wagner, 2007; Kolodziej, 2011; Careddu and Pettenazzo, 2018; Brendler et al., 2024). EPs^®^ 7630 (Dr. Willmar Schwabe GmbH & Co. KG, Karlsruhe, Germany) is a proprietary extract from the milled roots of *Pelargonium sidoides* (1:8–10), extraction solvent: ethanol 11% (w/w) (Schoetz et al., 2008) that was approved for the treatment of acute bronchitis in Germany in 2005 (Conrad et al., 2007b). In the meantime, EPs 7630-containing products have been approved in a number of countries in Europe, Asia, Australia, Central America, and South America for indications including acute bronchitis, common colds and acute respiratory tract infections, acute rhinosinusitis, and acute tonsillopharyngitis (Malek and Funk, 2024).

A broad range of constituents were identified in EPs 7630 and other *Pelargonium sidoides* extracts, including members of different compound classes such as amino acids, phenolic acids, α-hydroxy-acids, vitamins, polyphenols, flavonoids, coumarins, coumarins glucosides, coumarin sulphates, nucleotides, monomeric and oligomeric carbohydrates, minerals, peptides, purine derivatives, and highly substituted benzopyranones (Kolodziej, 2007; Schoetz et al., 2008; Moyo and Van Staden, 2014; Panara et al., 2022). This included gallocatechin- and epigallocatechin-based oligomeric and polymeric proanthocyanidins, phenolic and hydroxycinnamic acid-derivatives, highly oxygenated coumarins (7-hydroxy-5,6-di-methoxycoumarin; 6,8-dihydroxy-5,7-dimethoxycoumarin), gallic acid-derivatives, and the benzopyranones 6-methoxy-7-(sulfooxy)-2H-1-benzopyran-2-one, 6,8-bis(sulfooxy)-7-methoxy-2H-1-benzopyran-2-one, 7-hydroxy-6-methoxy-8-(sulfooxy)-2H-1-benzopyran-2-one, and 8-hydroxy-7-methoxy-6-(sulfooxy)-2H-1-benzopyran-2-one (Kolodziej, 2007; Theisen and Muller, 2012; Moyo and Van Staden, 2014).

Clinical trials have provided evidence of the efficacy of EPs 7630 against a range of acute respiratory infectious diseases. This includes placebo-controlled, randomized, double-blind trials in adult and pediatric patients with acute bronchitis and cough (Matthys et al., 2003; Chuchalin et al., 2005; Matthys and Heger, 2007a; Matthys and Funk, 2008; Kamin et al., 2010b; Kamin et al., 2010a; Matthys et al., 2010a; Kamin et al., 2012) and other clinical study formats (Blochin et al., 1999; Roots et al., 2004; Haidvogl and Heger, 2007; Matthys and Heger, 2007b; Matthys et al., 2007; Matthys et al., 2010b; Kardos et al., 2022). Moreover, a placebo-controlled, randomized, double blind clinical trial in healthy volunteers demonstrated a lack of pharmacological interaction between EPs 7630 and penicillin (Roots et al., 2004). A systematic review on herbal treatments in children identified EPs 7630 as the most commonly investigated one (Wopker et al., 2020). Moreover, a systematic overview with subgroup-analyses of clinical trial data for children younger than 6 years provided evidence of the effectiveness and safety of EPs 7630 in this age group (Kamin et al., 2018). A comprehensive review on safety and tolerability data from 29 clinical trials and non-interventional studies including a total of 10,026 patients showed EPs 7630 to be well tolerated in both children and adults (Matthys et al., 2013a). The safety of EPs 7630 in children was recently further confirmed in a clinical study (Kamin et al., 2023).

A number of additional studies provided clinical evidence of the efficacy of EPs 7630 against further respiratory infections including common cold (Lizogub et al., 2007; Keck et al., 2015; Riley et al., 2018; Riley et al., 2019), acute tonsillopharyngitis (Bereznoy et al., 2003; Bereznoj et al., 2009; Timen et al., 2015; Berezhnoi et al., 2016; Seifert et al., 2019), and acute rhinosinusitis (Schapoval and Heger, 2007; Bachert et al., 2009; Koch et al., 2016; Perić et al., 2020a). Moreover, EPs 7630 reduced the number of asthma attacks, cough frequency, and nasal congestion in asthmatic children with upper respiratory tract viral infections (Tahan and Yaman, 2013). In chronic obstructive pulmonary disease (COPD), EPs 7630 was investigated as an add-on therapy to a standardized baseline treatment (Matthys et al., 2013b). EPs 7630 increased the median time to exacerbation, reduced the number of exacerbations, reduced the use of antibiotics, improved quality of life, increased patient satisfaction, and reduced absences from work (Matthys et al., 2013b; Matthys and Funk, 2018). These findings resulted in the inclusion of EPs 7630 into the Swiss COPD treatment guidelines (Stolz et al., 2018).

Taken together, there is clinical evidence demonstrating that EPs 7630 is a safe and effective treatment for a range of acute infectious respiratory illnesses and that it can also improve conditions like COPD and asthma. Notably, EPs 7630 also has the potential to reduce the use of antibiotics (Matthys et al., 2013b; Perić et al., 2020a; Perić et al., 2020b; Martin et al., 2020), which is highly desirable given the threat posed by increasing levels of antibiotic resistance (Baran et al., 2023).

Given that EPs 7630 was shown to be beneficial for a range of clinical conditions (as outlined above) and has a complex composition (Schoetz et al., 2008), it is probably not surprising that many pharmacological activities have been described that may contribute to its therapeutic effects. Here, we provide an overview of the mechanisms of action that are likely to contribute to the clinical activity of EPs 7630 and other *Pelargonium sidoides* extracts, including the active ingredients if they are known.

This is a narrative review based on literature searches in PubMed (https://pubmed.ncbi.nlm.nih.gov) and Google Scholar (https://scholar.google.co.uk) using the search terms ‘*Pelargonium sidoides*’ and ‘EPs 7630’. In the following sections, we will give an overview of the known mechanisms that contribute to the antibacterial, interferon-inducing, antiviral, and immunomodulatory (beyond interferon induction) activities of EPs 7630 plus a section on additional mechanisms that may contribute to their beneficial effects against respiratory infections.

## 2 Pharmacological activities of EPs 7630 and other *Pelargonium sidoides* extracts

### 2.1 Antibacterial effects

An overview of the concentrations of EPs 7630, other *Pelargonium sidoides* extracts, and their constituents that displayed antibacterial effects is provided in Supplementary Table S1. In 1997, an initial study reported on effects of *Pelargonium sidoides* root extract against five Gram-negative (*Escherichia coli*, *Klebsiella pneumonia*, *Proteus mirabilis*, *Pseudomonas aeruginosa*, *Haemophilus influenzae*) and three Gram-positive bacteria (*Staphylococcus aureus*, beta-haemolytic *Streptococcus*, *Streptococcus pneumoniae*) (Kayser and Kolodziej, 1997). The study included five bacteria that commonly cause respiratory infections (*Klebsiella pneumonia*, *Haemophilus influenzae*, *Staphylococcus aureus*, beta-haemolytic *Streptococcus*, *Streptococcus pneumoniae*). Hydrolyzable tannins and low molecular weight phenols were identified as the main constituents of the extract (Kayser and Kolodziej, 1997). However, the antibacterial potency did not appear to explain the observed clinical effects on its own.

A number of further studies have in the meantime reported antibacterial effects of EPs 7630 against different bacteria. EPs 7630 inhibited *Helicobacter pylori* growth and with higher potency *Helicobacter pylori* adhesion to the gastric cancer cell line AGS and human stomach mucosa in a dose dependent manner (Beil and Kilian, 2007; Wittschier et al., 2007a; Wittschier et al., 2007b), with polymeric proanthocyanidins inhibiting *Helicobacter pylori* adhesion in a dose-dependent fashion (Wittschier et al., 2007b). Using HEp-2 and primary human buccal epithelial cells, it was shown that EPs 7630 inhibits group A *streptococcus* (GAS, *Streptococcus pyogenes*) adhesion to viable epithelial cells but also traps and inactivates bacteria by increasing their adhesion to dying and dead cells (Conrad et al., 2007c; Janecki and Kolodziej, 2010; Janecki et al., 2011). Proanthocyanidins and flavan-3-ols were shown to contribute to these anti-adhesive activities (Janecki and Kolodziej, 2010; Janecki et al., 2011).

One study suggested that *Pelargonium sidoides* extracts were active against the rapidly growing mycobacteria *Mycobacterium aurum* and *Mycobacterium smegmatis* that are usually non-pathogenic in humans, with oleic acid and linoleic acid being active fractions (Seidel and Taylor, 2004). Further research testing *Pelargonium sidoides* root extract against *Mycobacterium smegmatis* identified scopoletin, umckalin, catechin, and epigallocatechin as active ingredients (Mativandlela et al., 2007). Notably, the root extract was effective at lower concentrations than the investigated compounds, suggesting that its antimycobacterial activity is the consequence of the combination of different extract constituents. Moreover, the extract displayed some activity against human-pathogenic *Mycobacterium tuberculosis* strain TCC 27294 with a half maximal inhibitory concentration (IC50) of 2.5 mg/mL. Scopoletin, umckalin, catechin, and epigallocatechin did not affect *Mycobacterium tuberculosis* growth in the tested concentrations up to 200 μg/mL (Mativandlela et al., 2007). In contrast, EPs 7630 12.5 μg/mL reduced the growth of *Mycobacterium tuberculosis* strain H37Rv ATCC 27294 by 96% (Kolodziej et al., 2003). Further research showed that EPs 7630 inhibits *Mycobacterium tuberculosis* glycerol kinase and shikimate kinase (Lukman et al., 2020) and the neuraminidase of *Vibrio cholerae* and *Clostridium perfringens* (Quosdorf et al., 2017; Ochoa et al., 2017). In particular, the EPs 7630 tannin fractions with constituent *(epi)*gallocatechin/*(epi)*catechin units were found to inhibit bacterial neuraminidases (Quosdorf et al., 2017).

Proanthocyanidins isolated from *Pelargonium sidoides* root extracts displayed activity against *Porphyromonas gingivalis*, a periodontal and peri-implant pathogen, but not against the beneficial oral commensal *Streptococcus salivarius* (Savickiene et al., 2018). In a follow-up study on periodontal bacteria, the proanthocyanidin fraction of *Pelargonium sidoides* root extract displayed stronger antibacterial effects against *Staphylococcus aureus*, *Staphylococcus epidermidis*, and *Aggregatibacter actinomycetemcomitans* than against *Escherichia coli* (Jekabsone et al., 2019). Most recently, the combination of gallic acid and catechin, two constituents of EPs 7630, was active against 19 *Pseudomonas aeruginosa* clinical isolates and against biofilm formation (Abdel Bar et al., 2022).

However, it is not clear to which extent the antibacterial potency of *Pelargonium sidoides* contributes to the beneficial clinical effects reported for EPs 7630 (Kayser and Kolodziej, 1997; Kolodziej et al., 2003). Two clinical trials compared the efficacy of EPs 7630 and antibiotics for the treatment of uncomplicated acute bacterial rhinosinusitis: One study reported significant symptom improvements by both EPs 7630 and antibiotic therapy vs. the control group, albeit with superior clinical activity of roxithromycin over EPs 7630 (Perić et al., 2020c). The other reported higher clinical and antibacterial efficacy of EPs 7630 relative to amoxicillin (Perić et al., 2020a). In the latter study, cultivation experiments using middle meatal samples found fewer types of bacteria in the cultures from EPs 7630 patients than from amoxicillin patients (Perić et al., 2020a).

**FIGURE 1:**
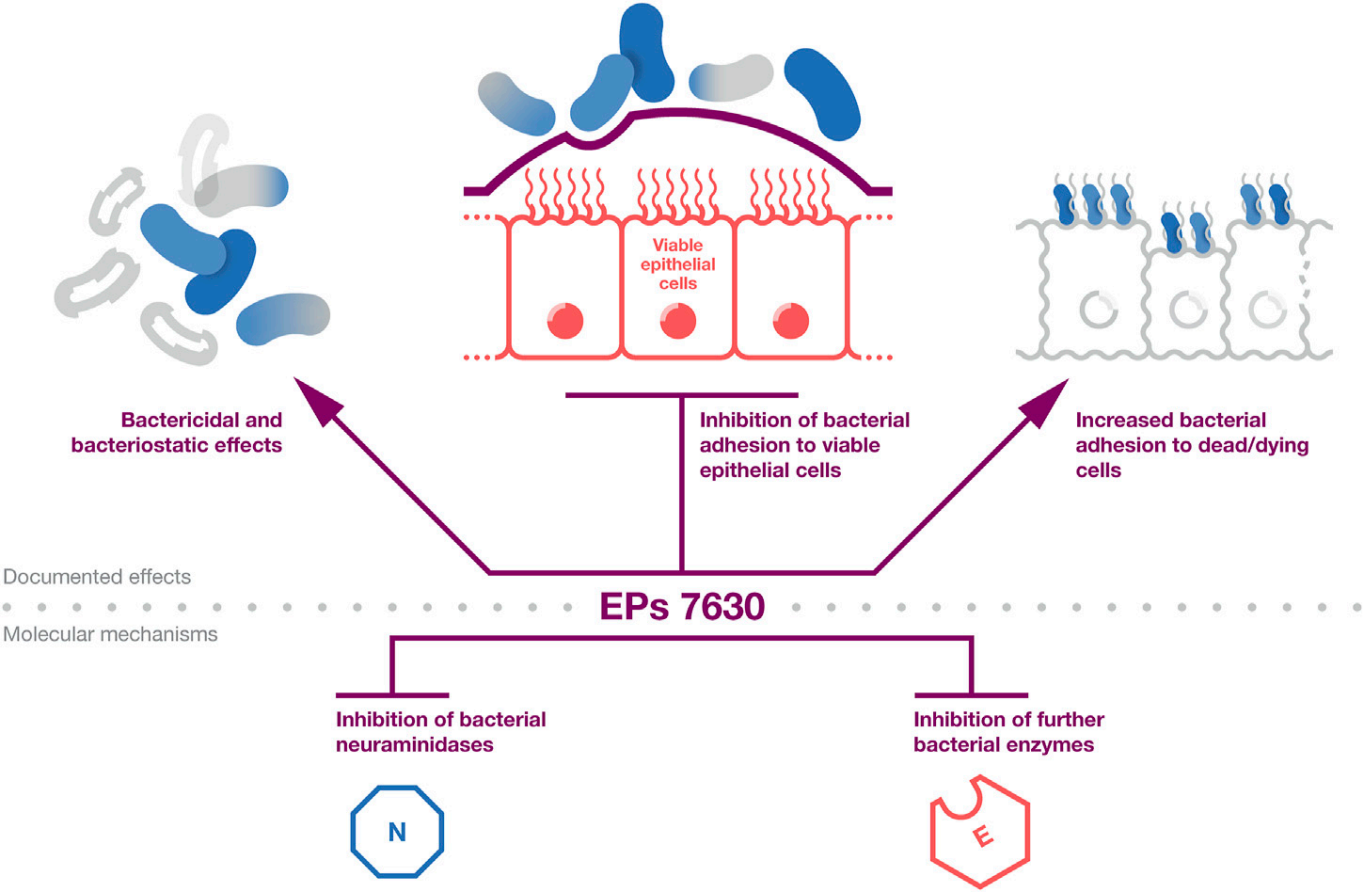
Antibacterial effects. A summary of the evidence on the antibacterial activity of EPs 7630. The top part provides the documented effects that affect bacterial infection and viability. The bottom part provides documented antibacterial mechanisms that are likely to contribute to the effects outlined in the top part.

The documented antibacterial effects of EPs 7630 and underlying mechanisms are summarized in Figure 1. Taken together, EPs 7630s antibacterial effects, which are mediated by ingredients including proanthocyanidins, gallic acid, and catechin, are likely to contribute among additional mechanisms to the extract’s clinical activity in patients with bacterial respiratory tract infections.

### 2.2 Induction of an interferon response

The vast majority of upper respiratory tract infections commonly designated as common cold are caused by viruses including rhinoviruses, coronaviruses, influenza viruses, adenoviruses, respiratory syncytial virus, and parainfluenza virus (Gonzales and Sande, 2000; Fendrick et al., 2003; Jartti et al., 2012; Jacobs et al., 2013; Moriyama et al., 2020).

The first indication that *Pelargonium sidoides* extracts may exert antiviral effects was provided by a study reporting interferon-like activity of gallic acid, a constituent of *Pelargonium sidoides* extract (Kayser et al., 2001). The interferon response is an intracellular innate immune reaction that is triggered when so-called ‘pathogen-associated molecular patterns’ (PAMPs) are detected by so-called ‘pattern recognition receptors’ (PRRs) (Minkoff and tenOever, 2023). The activation of pattern recognition receptors results in the secretion of interferons that induce an innate immune response in a paracrine and autocrine fashion and are critical for the induction of an adaptive immune response (Parker and Prince, 2011; Lazear and Schoggins, 2019; Minkoff and tenOever, 2023).

Gallic acid protected murine L929 cells from lysis by encephalomyocarditis virus (cardiovirus A) that belongs to the picornavirus family, which also includes rhinoviruses, the most frequent cause of common colds (Kayser et al., 2001). Later studies demonstrated that EPs 7630 induces comparable interferon-like effects (Kolodziej et al., 2003; Thäle et al., 2011). Moreover, interferon-like activity was detected in the supernatants of EPs 7630-treated murine macrophages, and EPs 7630 increased the polyinosinic acid:polycytidylic acid (pI:pC)-induced interferon-beta production in MG-63 cells (Kolodziej et al., 2003; Thäle et al., 2011).

**FIGURE 2:**
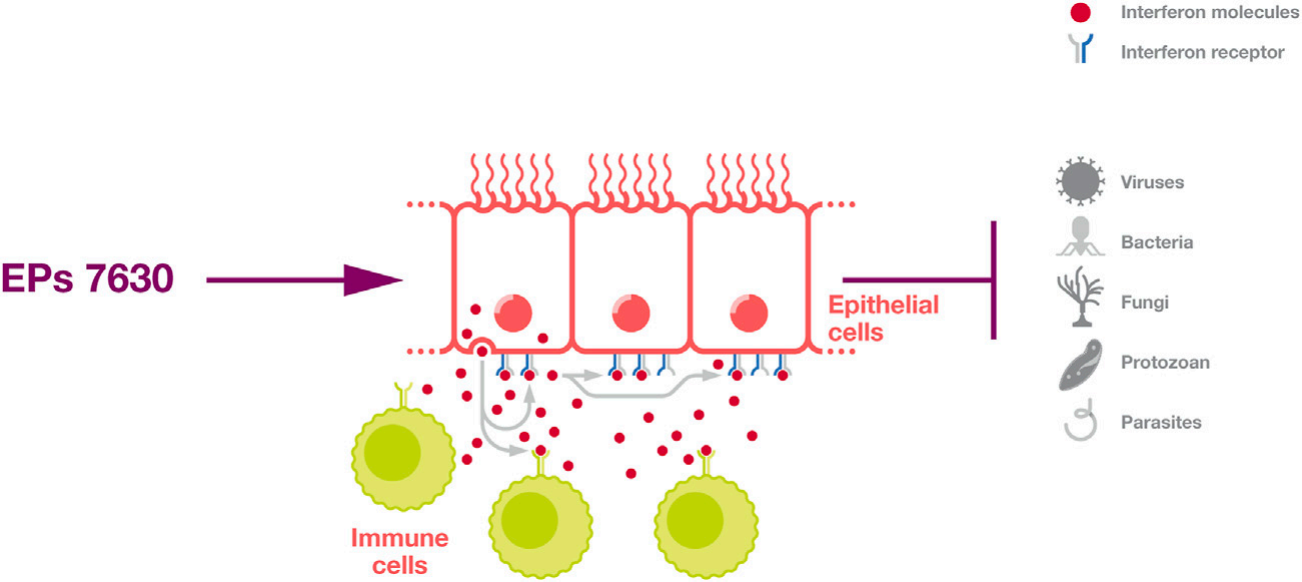
Induction of an interferon response. EPs 7630 can induce an interferon response that protects cells in an autocrine and paracrine fashion and activates immune cells.

Further, interferon signalling provides protection against a broad range of pathogens (Figure 2) and plays a fundamental role in the control of many infectious diseases (Parker and Prince, 2011; Lazear and Schoggins, 2019; Minkoff and tenOever, 2023). For example, individuals with defects in interferon signaling are highly vulnerable to severe COVID-19 and COVID-19-associated death (Bastard et al., 2020; Zhang et al., 2020; Minkoff and tenOever, 2023).

### 2.3 Antiviral effects

An overview of the concentrations of EPs 7630, other *Pelargonium sidoides* extracts, and their constituents that displayed antiviral effects is provided in Supplementary Table S2. An investigation using a panel of respiratory viruses showed that EPs 7630 concentrations of up to 100 μg/mL inhibited virus-induced cytopathogenic effect formation in cells infected with H1N1 and H3N2 influenza A viruses, respiratory syncytial virus (RSV), the common cold coronavirus HCo-229E, parainfluenzavirus 3, and coxsackie A9 virus (but not in cells infected with H5N1 influenza A virus, adenovirus 3 and 7, or rhinovirus 16) (Michaelis et al., 2011). The antiviral effects against seasonal influenza A viruses, RSV, HCo-229E, parainfluenzavirus, and coxsackie A9 virus were confirmed by the determination of infectious virus titers (Michaelis et al., 2011). Notably, a secondary subgroup analysis of an open-label uncontrolled clinical trial (Keck et al., 2015) suggested that EPs 7630 is active against common cold human coronaviruses in patients (Keck et al., 2021).

A subsequent study confirmed that EPs 7630 is active against H1N1 influenza A viruses but not against adenovirus (and measles virus) (Theisen and Muller, 2012). Time-of-addition experiments suggested that EPs 7630 interferes with influenza virus entry into host cells. Notably, a previous study had reported that an aqueous *Pelargonium sidoides* extract inhibited herpes simplex virus type 1 (HSV-1) cellular entry into RC-37 cells (Schnitzler et al., 2008).

Moderate effects were also detected on influenza virus attachment to host cells and viral neuraminidase activity (Theisen and Muller, 2012). Inhibition of H1N1 influenza A virus neuraminidase was later confirmed in another study that showed that EPs 7630 synergistically enhanced the activity of zanamivir, a neuraminidase inhibitor that is approved for the treatment of influenza viruses (Quosdorf et al., 2017). Gallocatechin- and epigallocatechin-based oligo- and polymeric polyphenol fractions were identified as the most active ingredients (Theisen and Muller, 2012; Quosdorf et al., 2017). Moreover, EPs 7630 inhalation protected mice from H1N1-induced mortality (Theisen and Muller, 2012). In agreement, another *Pelargonium sidoides* root extract was also found to exert anti-influenza A virus activity, to inhibit influenza A virus cell entry, and to inhibit the influenza A virus neuraminidase (Walther et al., 2020). Polyphenolic constituents of EPs 7630, in particular secondary metabolites such as flavonoids and leuco-anthocyanidins, were also found to inhibit HIV attachment to and infection of peripheral blood mononuclear cells and macrophages (Helfer et al., 2014).

There is inconclusive evidence on the effects of EPs 7630 and other *Pelargonium sidoides* root extracts on rhinoviruses. In disagreement with the originally reported lack of activity of EPs 7630 against rhinovirus (Michaelis et al., 2011), a later study described EPs 7630-mediated rhinovirus inhibition as determined by cytopathogenic effect formation, immunofluorescence, and rhinovirus RNA detection by PCR (Roth et al., 2019), while a report on another *Pelargonium sidoides* root extract did also not detect anti-rhinovirus activity (Walther et al., 2020). The differences may be explained by the use of different experimental systems. The EPs 7630 studies used different rhinovirus 16 strains (Michaelis et al., 2011; Roth et al., 2019), while the third study used rhinovirus A2 and B14 (Walther et al., 2020). Moreover, the first EPs 7630 study investigated rhinovirus 16-induced cytopathogenic effect formation in the melanoma cell line Mel-Ho (Michaelis et al., 2011). The second EPs 7630 study quantified virus-infected primary human bronchial epithelial cells (Roth et al., 2019). Finally, the study of the alternative *Pelargonium sidoides* root extract measured the rhinovirus-induced cytopathogenic effects in HeLa Ohio cells (Walther et al., 2020). The study that reported anti-rhinovirus activity of EPs 7630 speculated that the observed anti-rhinovirus effects may be the consequence of the downregulation of host cell proteins that are involved in virus attachment and of the upregulation of host cell defense proteins (Roth et al., 2019). Hence, it is plausible that EPs 7630 may exert different effects on different cell types, which may then result in different susceptibility to rhinovirus infection. In agreement, a further study suggested that upregulation of the vitamin D receptor on host cells, another host cell-specific mechanism, contributes to the anti-rhinovirus effects of EPs 7630 (Roth et al., 2021). Recent findings additionally showed that EPs 7630 reduces the levels of the rhinovirus receptor ICAM and the virus-binding tight junction proteins desmoglein2, desmocollin2, ZO-1, claudin1, and claudin4 on airway epithelial cells (Fang et al., 2023).

The effects of EPs 7630 against SARS-CoV-2 and other human-pathogenic coronaviruses were also investigated (Papies et al., 2021; Alossaimi et al., 2022; Emanuel et al., 2023). EPs 7630 reduced virus titres of MERS-CoV, SARS-CoV, and SARS-CoV-2 in both interferon-competent Calu-3 and interferon-deficient VeroFM cells (Papies et al., 2021). The EPs 7630 IC50 values for an original D614 SARS-CoV-2 strain were 0.48 μg/mL in VeroFM and 1.61 μg/mL in Calu-3 cells, indicating that interferon-independent effects significantly contribute to the anti-SARS-CoV-2 activity of EPs 7630. The efficacy of EPs 7630 against Alpha and Beta SARS-CoV-2 strains was lower than against the D614 strain. In control experiments, EPs 7630 also inhibited Rift Valley fever virus at a concentration of 100 μg/mL but not mumps virus (Papies et al., 2021).

In a subsequent study, EPs 7630 exerted only moderate effects against an early SARS-CoV-2 isolate (BetaCoV/Munich/BavPat1/2020, B.1) in human bronchial airway epithelial cells in air-liquid interface cultures (Emanuel et al., 2023). Further experiments showed that EPs 7630 displayed more pronounced effects against a SARS-CoV-2 Omicron isolate than against B.1 and a Delta isolates, both in nasal and bronchial human epithelial cells, thereby exerting stronger effects in bronchial human epithelial cells (Emanuel et al., 2023).

SARS-CoV-2 host cell entry is mediated by the interaction of the viral spike (S) protein with its cellular receptor ACE2 and depends on S cleavage by host cell proteases such as TMPRSS2 and cathepsin L (Jackson et al., 2022). TMPRSS2 is located and can activate S on the cell surface, resulting in direct membrane fusion of ACE2-bound SARS-CoV-2. Cathepsin L cleaves S after receptor-mediated endocytosis in the endolysosome (Jackson et al., 2022). EPs 7630 inhibited the entry of SARS-CoV-2 S-carrying pseudovirus particles in TMPRSS2-positive Calu-3 and TMPRSS2-negative VeroFM cells. Moreover, EPs 7630 inhibited SARS-CoV-2 entry in Calu-3 and TMPRSS2-negative VeroE6 cells as indicated by the levels of subgenomic SARS-CoV-2 nucleopcapsid (N) RNA (Papies et al., 2021).

Subsequent experiments demonstrated that EPs 7630 is particularly effective against Omicron viruses that are predominantly internalized by endocytosis (Emanuel et al., 2023). Together, these data indicate that EPs 7630 can interfere both with membrane fusion- and endocytosis-mediated SARS-CoV-2 entry and that it may particularly interfere with the endosomal uptake of Omicron variant viruses (Papies et al., 2021; Emanuel et al., 2023). Notably, Omicron viruses have also been suggested to be less effective at antagonizing host cell interferon responses and more sensitive to interferon treatment than other variants (Bojkova et al., 2022a; Bojkova et al., 2022b; Bojkova et al., 2022c; Hu et al., 2022; Shuai et al., 2022; Bojkova et al., 2023). Hence, EPs 7630-induced interferon induction may also have contributed to its anti-Omicron activity.

The investigation of sub-fractions of EPs 7630 with different molecular weights revealed that lower molecular weight fractions were more effective at inhibiting virus entry but that larger molecular weight fractions were more effective at inhibiting virus replication (Papies et al., 2021). A second study confirmed the activity of EPs 7630 against a SARS-CoV-2 D614 in VeroE6 cells, but the reported IC50 was higher (13.8 μg/mL) (Alossaimi et al., 2022) than in the previous one (Papies et al., 2021). The discrepancy may be explained by the use of different assays for the IC50 determination. The initial study calculated the IC50 for the reduction in virus titers (Papies et al., 2021), whereas the second one quantified the cytopathogenic effect (Alossaimi et al., 2022). In the second study, the EPs 7630 ingredient scopoletin was proposed to contribute to the observed antiviral effects with an IC50 of 17.79 μM (Alossaimi et al., 2022). A follow-up study identified the known EPs 7630 constituent epigallocatechin and taxifolin as well as the putative EPs 7630 constituent epigallocatechin gallate as molecules with anti-SARS-CoV-2 activity (Emanuel et al., 2023).

**FIGURE 3:**
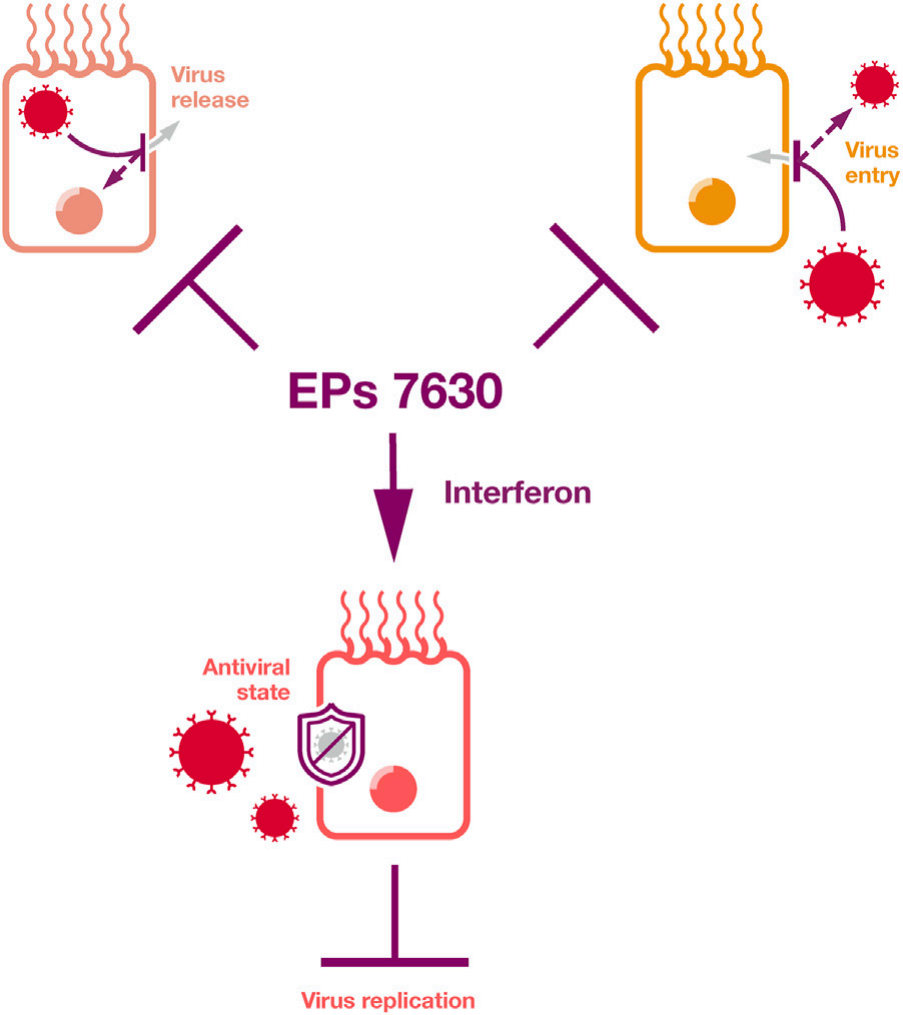
Antiviral effects. EPs 7630 inhibits virus replication by the inhibition of virus release, by the inhibition of virus attachment and entry, by inducing an interferon response, and by additional mechanisms that will need to be elucidated by future research.

Taken together, EPs 7630 can interfere with the replication of a range of viruses by inducing an interferon response, by virus entry inhibition, by virus release inhibition, and by further mechanisms that will have to be elucidated in future studies (Figure 3). Ingredients known to be involved in these antiviral activities include proanthocyanidins, scopoletin, epigallocatechin, epigallocatechin gallate, and taxifolin, as well as gallic acid as an inducer of an interferon response.

### 2.4 Immunomodulatory effects

In addition to the induction of interferon signaling, a pathway involved in the innate immune response (Minkoff and tenOever, 2023), EPs 7630 has been reported to exert further immunomodulatory effects. An initial study showed that *Pelargonium sidoides* root extract protected murine macrophages from infection with the obligate intracellular protozoan *Leishmania donovani* without exerting direct anti-protozoan effects (Kayser et al., 2001). The extract caused interferon-like effects (see above) and macrophage activation as indicated by TNF-alpha and nitric oxide (NO) induction. Gallic acid and its methyl ester were postulated as the main active ingredients. Interestingly, NO induction was not found to be critically involved in the *Pelargonium sidoides*-mediated effects against intracellular *Leishmania donovani* (Kayser et al., 2001).

Additional studies showed that EPs 7630 (and gallic acid as an active ingredient) also causes the activation of *Leishmania donovani*- and *Listeria monocytogenes*-infected macrophage as indicated by TNF-alpha and NO production, with some level of specificity for infected over non-infected cells (Kolodziej et al., 2003; Trun et al., 2006; Thäle et al., 2008; Thäle et al., 2011). In agreement, EPs 7630 also increased *Candida albicans* phagocytosis as well as oxidative burst and intracellular killing by human peripheral blood phagocytes (Conrad et al., 2007a).

In a follow-up study, EPs 7630 induced the expression of IL-1, IL-12, IL-18, TNF, IFN-alpha, IFN-gamma, and iNOS in *Leishmania major*-infected RAW 264.7 cells (an Abelson leukaemia virus-transformed cell line derived from BALB/c mice often used as a macrophage model) but not in non-infected cells (Trun et al., 2006). These findings provided further insights into the molecular processes associated with EPs 7630-induced macrophage activation and suggested that such effects may be specific for infected cells. Again, gallic acid was identified as one of the potential active EPs 7630 ingredients (Trun et al., 2006). In agreement, EPs 7630 increased IL-1 and IL-12 production in *Listeria monocytogenes*-infected primary mouse macrophages, with the effects on IL-12 being more pronounced in infected than in non-effected cells (Thäle et al., 2008). Moreover, IL-1 and IL-12 secretion was specifically enhanced by EPs 7630 in infected macrophages relative to non-infected macrophages. EPs 7630 also increased CD40 levels on *Listeria monocytogenes*-infected macrophages, which bind to CD40-L on CD4^+^ T-cells and mediate a CD4^+^ T-cell immune response (Thäle et al., 2008).

A study using primary peripheral blood mononuclear cells found that EPs 7630 increased the secretion of proinflammatory TNF-alpha, proinflammatory IL-6, and antiinflammatory IL-10. The EPs 7630-induced cytokine profile differed from that induced by TLR3 ligands, TLR4 ligands, an interleukin cocktail (IL-1beta, IL-2, IL-12), anti-CD3 antibodies, and anti-CD28 antibodies (Witte et al., 2015). Further research revealed that EPs 7630 activated monocytes, predominantly via the MAP kinases JNK1/2, p38, and ERK1/2. Interestingly, EPs 7630 reduced TLR3 ligand (model for viral infection)- and TLR4 ligand (model for bacterial infection)-induced TNF-alpha and IL-10 production but induced high levels of IL-6 (Witte et al., 2015). IL-6 is known to differentiate CD4^+^ T cells into T helper 17 (Th17) and T helper 22 (Th22) cells, which are both involved in pathogen clearance (Chou, 2013).

In agreement, a subsequent study found that the incubation of primary human peripheral blood mononuclear cells with EPs 7630 resulted in the induction of IL-17 (indicates Th17 differentiation), IL-22 (indicates Th22 differentiation), and interferon-gamma (Witte et al., 2020). In combination with T cell-stimulating anti-CD3 and anti-CD28 antibodies, EPs 7630 increased IL-17 and IL-22 levels but not interferon-gamma levels. This indicates that EPs 7630 does not non-specifically upregulate the secretion of all cytokines by T cells (Witte et al., 2020).

Experiments using A549 cells (a lung cancer cell line used as a model for lung tissue) and mouse experiments showed that IL-22 increased S100A9 protein levels in airway tissues (Witte et al., 2020). S100A9 is an antimicrobial protein that protects epithelial barriers from invasion by pathogens including bacteria, viruses, fungi, and protozoa (Johnstone and Herzberg, 2022). These findings agree with previous experiments showing that IL-22 and IL-17 increase the expression of S100A9 (Liang et al., 2006).

Further research indicated that the EPs 7630-induced IL-22 induction is the consequence of monocyte activation and depends on the combined effects of paracrine T cell stimulation by monocyte-derived cytokines including IL-1 and IL-23 and direct monocyte-CD4^+^ T cell contact (Witte et al., 2020).

In addition to immunomodulatory actions that activate the innate immune system, EPs 7630 has also been described to control overshooting inflammation that can contribute to the pathogenesis caused by respiratory pathogens (Perić et al., 2020b; Perić et al., 2020c; Papies et al., 2021). Two clinical studies investigated chemokine levels in the nasal secretions of patients with acute postviral rhinosinusitis and uncomplicated acute bacterial rhinosinusitis (Perić et al., 2020b; Perić et al., 2020c). They found increased levels of chemokines including MCP-1, IP-10, and MIP-1beta and decreased levels of MIP-1alpha, ENA-78, GROalpha, and IL-8 in EPs 7630-treated patients (Perić et al., 2020b; Perić et al., 2020c). Given the complex, context- and cell type-dependent actions and interactions of such cytokines (Verbeke et al., 2012; Wang, 2018; Korchagina et al., 2021; Kolter et al., 2022), there is no straightforward interpretation of these findings. However, the observed EPs 7630-induced chemokine profiles were associated with improved clinical performance in both studies (Perić et al., 2020b; Perić et al., 2020c). Hence, it can be speculated that the EPs 7630-induced increased MCP-1 and IP-10 levels are associated with a protective innate immune response mediated by monocyte attraction (Dufour et al., 2002; Verbeke et al., 2012; Vazirinejad et al., 2014; Bianconi et al., 2018; Perić et al., 2020b), whereas reduced ENA-78, GROalpha, and IL-8 levels may prevent neutrophil-mediated tissue damage (Verbeke et al., 2012; Wang, 2018; Perić et al., 2020b).

Further studies provided evidence that *Pelargonium sidoides* extracts can exert immunomodulatory effects that both strengthen the immune response and prevent an overactivation of the immune system. In a clinical study in athletes performing intensive running sessions, a *Pelargonium sidoides* extract prevented the exercise-associated reduction in potentially protective secreted immunoglobulin A levels and reduced in saliva the levels of proinflammatory IL-6 and IL-15, which may be associated with aberrant inflammatory reactions in response to exercise (Luna et al., 2011).

Moreover, a combined *Pelargonium sidoides*/*Coptis chinensis* (2:1 ratio) extract inhibited the production of pro-inflammatory molecules (NO, PGE2, TNF-alpha, IL-1beta, IL-6) in lipopolysaccharide (LPS)-treated RAW 264.7 cells, which was anticipated to be the consequence of suppression of NFkappaB signalling (Park et al., 2018). In agreement, EPs 7630-mediated NF-kappaB inhibition was also described in head and neck squamous cell carcinoma cell lines (Meyer et al., 2011). The *Pelargonium sidoides*/*Coptis chinensis* extract also reduced carrageenan-induced oedema as well as mast cell degranulation and the production of inflammatory mediators (COX-2, iNOS, TNF-alpha) in a rat paw model (Park et al., 2018).

Anti-inflammatory effects were also described for a *Pelargonium sidoides*/*Coptis Rhizoma* (2:1 ratio) extract (Min et al., 2020). The combined extract exerted beneficial effects in an ovalbumin-induced asthma model in C57BL/6J mice, which reflects allergic asthma. In contrast to the corticosteroid dexamethasone, which was used as a positive control, *Pelargonium sidoides*/*Coptis Rhizoma* extract treatment was not associated with a loss of body weight as adverse side-effect (Min et al., 2020). These findings agree with the anti-asthma effects determined for EPs 7630 in clinical trials (Tahan and Yaman, 2013).

In a study focusing on periodontitis-related conditions, a *Pelargonium sidoides* root extract and its proanthocyanidin fraction displayed anti-inflammatory and tissue-protective activities (Jekabsone et al., 2019). Both, the extract and the proanthocyanidin fraction, protected primary murine gingival fibroblasts from necrosis and apoptosis induced by bacterial LPS. In addition, they reduced the release of IL-8 and PGE2 from LPS-treated primary murine gingival fibroblasts and of IL-6 from primary human PBMCs (Jekabsone et al., 2019). *Pelargonium sidoides* root extract and its proanthocyanidin fraction also decreased IL-1beta and iNOS mRNA levels in LPS/interferon-gamma-stimulated primary murine bone marrow-derived macrophages and IL-1beta and COX-2 mRNA levels in LPS-activated primary human PBMCs. Additionally, both preparations prevented murine macrophage polarisation into pro-inflammatory M1 macrophages associated with periodontitis, as indicated by a suppression of CD80 and CD86 cell surface presentation in response to LPS/interferon-gamma treatment (Jekabsone et al., 2019).

Immunomodulatory effects of EPs 7630 were also observed in SARS-CoV-2-infected cells (Papies et al., 2021; Emanuel et al., 2023). Notably, severe life-threatening COVID-19 is the consequence of an overshooting immune response, a so-called cytokine storm (Brendler et al., 2021; Kaivola et al., 2021). In Calu-3 cells, EPs 7630 reduced the SARS-CoV-2-mediated secretion of a range of pro-inflammatory mediators known to contribute to cytokine storm including TNFalpha, IL-4 IL-8, IL-13, CXCL9, IP-10/CXCL10, PDGF, VEGF-A, and CD40L (Brendler et al., 2021; Kaivola et al., 2021; Papies et al., 2021; Petrey et al., 2021; Zhu et al., 2022; Mohseni Afshar et al., 2023; Ng et al., 2023). In agreement with previous findings, IL-6 was the only investigated prominent proinflammatory mediator that was consistently upregulated (Witte et al., 2015; Papies et al., 2021; Ng et al., 2023). The relevance of this remains to be further investigated.

Moreover, multiple immunoregulatory molecules including TNFAIP3, IL-9, IL-12, and IL-15 were upregulated by EPs 7630, suggesting that EPs 7630 may on balance counteract the development of a SARS-CoV-2-induced cytokine storm (Papies et al., 2021). Similar immunomodulatory effects by EPs 7630 were detected in SARS-CoV-2 (B.1)-infected human bronchial airway epithelial cell air-liquid interface cultures (Emanuel et al., 2023). Notably, the immunomodulatory effects mediated by EPs 7630 were at least in part separated from the antiviral effects. EPs 7630 fractions with limited antiviral activity displayed pronounced immunomodulatory activity in SARS-CoV-2 (B.1)-infected human bronchial airway epithelial cell air-liquid interface cultures (Papies et al., 2021; Emanuel et al., 2023). This suggests that the different ingredients of the extract exert complementary effects that contribute to the beneficial effects of EPs 7630 observed for respiratory illnesses.

A subsequent study of the EPs 7630-mediated effects on COVID-19 in a SARS-CoV-2-infected hamster model showed that EPs 7630 modulated the COVID-19 pathology predominantly by immunomodulatory effects (Emanuel et al., 2023).

**FIGURE 4:**
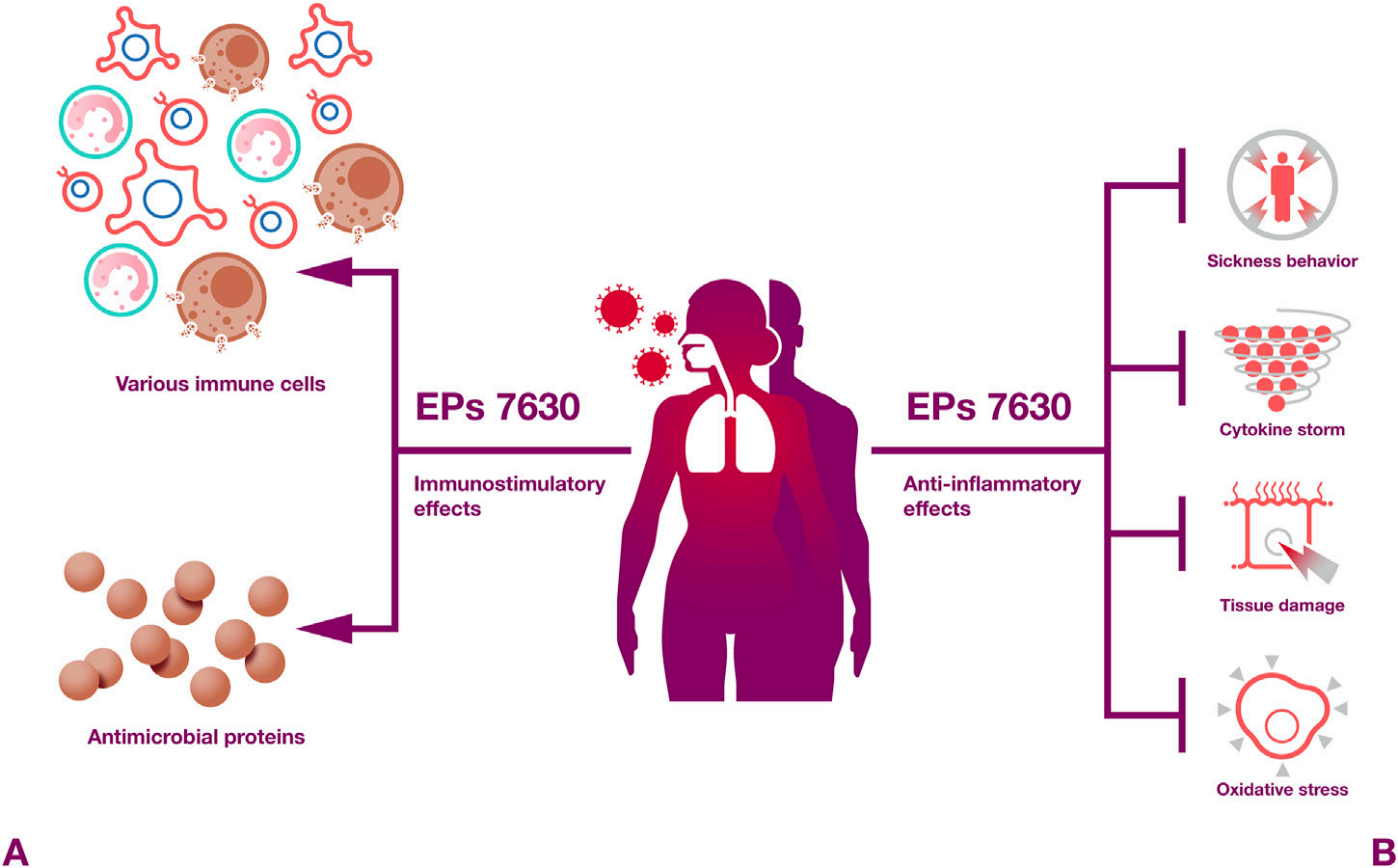
Immunomodulatory effects. EPs 7630 induces both protective immunostimulatory and anti-inflammatory effects.

Taken together, EPs 7630 and *Pelargonium sidoides* root extracts in general exert numerous immunomodulatory activities (Figure 4) that are likely to contribute to the beneficial effects of EPs 7630 observed against respiratory illnesses in clinical trials. In particular, proanthocyanidins and gallic acid have been described as pharmacolocigally active constituents that exert immunomodulatory effects.

### 2.5 Additional mechanisms

In addition to the described antibacterial, antiviral, and immunomodulatory effects, EPs 7630 has been shown to induce further pharmacological effects that are likely to contribute to its activity against respiratory illnesses.

#### 2.5.1 Improved tissue barrier function

**FIGURE 5:**
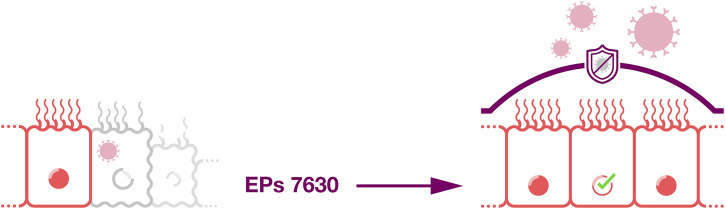
Improved tissue barrier function. EPs 7630 may improve the barrier function of airway epithelium by inducing epithelial cell replication and wound healing and by reducing virus attachment.

A recent study using primary airway epithelial cells and the immortalized airway epithelial cell line NuLi-1 showed that EPs 7630 protects airway epithelial cells and counteracts rhinovirus-induced damage (Fang et al., 2023). EPs 7630 increased airway epithelial cell proliferation and wound healing in a scratch assay in the absence and presence of rhinovirus. Moreover, EPs 7630 shifted the extracellular matrix composition in rhinovirus-infected cell cultures towards a non-inflammatory phenotype. EPs 7630 further downregulated virus-binding tight junction proteins. Hence, these results suggest that EPs 7630 improves the barrier function of airway epithelium by inducing epithelial cell replication and wound healing and by reducing virus attachment (Fang et al., 2023; Figure 5). Mechanistically, these effects were at least in part mediated via cAMP/Akt and cAMP/p38MAPK signaling (Fang et al., 2023).

Since the airway epithelium represents a critical first line defense against infection (Jakwerth et al., 2022), the protective effects exerted by EPs 7630 observed on airway epithelial cells (Fang et al., 2023) are likely to contribute to the clinical effects observed for EPs 7630 against respiratory diseases.

#### 2.5.2 Sickness behavior

The term ‘sickness behavior’ describes a set of physiological and behavioral changes in response to infection and inflammation as a protection mechanism. Symptoms include fever, malaise, lethargy, loss-of-appetite, and fatigue and are thought to be caused by systemic and local cytokine secretion (Krapić et al., 2021). Hence, EPs 7630 may impact on sickness behavior by causing anti-inflammatory effects, as described in the previous section.

Indeed, EPs 7630 inhibited sickness behaviour in LPS-treated mice as indicated by the so-called “light-dark-box” method (Nöldner and Schötz, 2007). When mice are first placed into a box with a white and a black compartment, naïve mice usually prefer the dark part. LPS treatment causes proinflammatory cytokine production. This results in sickness behavior characterized by reduced mouse movement between the compartments and a higher fraction of time spent in the (normally avoided) light compartment. The investigation of different EPs 7630 fractions showed that the high molecular weight fraction (>30 kDa) is predominantly responsible for the EPs 7630-induced reduction in LPS-induced sickness behavior (Nöldner and Schötz, 2007). Follow-up research indicated that amino-substituted (epi)gallocatechin oligomers from EPs 7630 inhibit LPS-induced sickness behavior in mice but did not detect significant effects of EPs 7630 fractions on motility, body weight, body temperature, motor coordination, convulsions, or central analgesia in healthy animals (Schötz and Nöldner, 2007; Schötz et al., 2019). This indicates that the observed behavioral effects are limited to sick animals.

#### 2.5.3 Antitussive effects

The antitussive effects of EPs 7630 were investigated in two different animal models; in an ammonia-induced cough model in mice and in a citric acid-induced cough model in guinea pigs (Bao et al., 2015). EPs 7630 significantly reduced the number of coughs in both models (Bao et al., 2015).

#### 2.5.4 Secretolytic and secretomotoric effects

The mucociliary system protects the airways from pathogens and foreign particles as a physical barrier (Pangeni et al., 2023). Mucus produced by secretory cells is transported by ciliated cells out of the airways (Pangeni et al., 2023). Initial evidence that EPs 7630 promotes airway clearance stemmed from a study showing that the extract increased the ciliary beat frequency in a monolayer of primary human ciliated cells derived from the nasal epithelium (Neugebauer et al., 2005). These findings were confirmed *in vivo* by a study showing that EPs 7630 increases the clearance of phenol red from the airways of mice in a dose-dependent manner (Bao et al., 2015). Notably, these secretolytic effects may contribute to the observed antitussive effects (Bao et al., 2015).

#### 2.5.5 Inhibition of oxidative stress

EPs 7630 was shown to prevent lung damage and reduce inflammation in an acute bacterial bronchitis model in rats induced by daily inhalation of unburned smoke over a period of 21 days and weekly intranasal applications of *Streptococcus pneumoniae* (Bao et al., 2015). Antioxidative effects of EPs 7630 appear to have contributed to these therapeutic effects as indicated by the upregulation of superoxide dismutase, an important endogenous antioxidant, and the downregulation of malondialdehyde, a marker of oxidative stress levels (Bao et al., 2015).

EPs 7630 also exerted antioxidative effects in a further study (Rezaizadehnajafi and Wink, 2014). It reduced the free radical 2,2-Diphenyl-1-picrylhydrazyl (DPPH*) with an IC50 of 14.7 μg/mL, as indicated by a reduction in 2,2-Diphenyl-1-picrylhydrazyl fluorescence. Moreover, EPs 7630 reduced oxidative stress-induced GFP expression in juglone (produces superoxides)-treated *Caenorhabditis elegans* hsp-16.2::GFP(gpIs1) mutants and protected *Caenorhabditis elegans* from a lethal juglone dose (Rezaizadehnajafi and Wink, 2014). Mechanistic studies suggested that EPs 7630 exerts its protective effects at least in part by the activation of the transcription factor DAF-16/FOXO via insulin/IGF-1 signalling (Rezaizadehnajafi and Wink, 2014).

#### 2.5.6 Antifungal and antiparasitic effects

Fungal pathogens and parasites are rarely involved in respiratory infections (Araujo et al., 2024; Ling et al., 2024). Nevertheless, it is worth mentioning that EPs 7630 has also been shown to exert antifungal effects and antiparasitic activity beyond the previously mentioned anti-leishmanial activity.

Root extracts derived from *Pelargonium sidoides* roots inhibited the growth of *Aspergillus niger* and *Fusarium oxysporum* (Mativandlela et al., 2006), and a *Pelargonium sidoides* leaf extract inhibited the growth of *Candida albicans* (Moyo et al., 2013). Moreover, the *Pelargonium sidoides* tubers have been shown to contain compounds with known antifungal activity including gallic acid, scopoletin, fraxetin, catechin, proanthocyanidins, and β-sitosterol, and a *Pelargonium sidoides* tuber extract was shown to inhibit *Cryptococcus neoformans* growth (Samie et al., 2019). EPs 7630 furthermore increased the phagocytosis and intracellular killing of *Candida albicans* by human peripheral blood phagocytes (Conrad et al., 2007a).

Kalobin^®^, an extract from the roots of *Pelargonium reniforme* and *sidoides* was found to reduce the infection of mice with the trematodes *Prohemistomum vivax* and *Schistosoma mansoni* and the nematode *Trichinella spiralis* (Amer et al., 2006; Abdel Menaem et al., 2022; Soliman et al., 2023).

## 3 Discussion

Based on the available evidence, a wide range of mechanisms appears to contribute to the beneficial effects observed for EPs 7630 in the treatment of infectious and inflammatory respiratory illnesses. These mechanisms include antibacterial, antiviral, immunomodulatory, tissue-protective, and secretolytic/secretomotoric activity, as well as enhancing epithelial barrier integrity. Given the broad range of clinical activities and documented pharmacological effects of EPs 7630, it is likely that multiple mechanisms, which are at least in part exerted by different constituents of the extract, may contribute to the observed therapeutic profile.

There is a limited understanding of which active ingredients are responsible for which effects. Proanthocyanidins have been shown to contribute to the antibacterial (Kayser and Kolodziej, 1997; Wittschier et al., 2007b; Savickiene et al., 2018; Jekabsone et al., 2019), antiviral (Theisen and Muller, 2012; Quosdorf et al., 2017), and immunomodulatory activities (Jekabsone et al., 2019) of *Pelargonium sidoides* extracts. Gallic acid exerts antibacterial and immunomodulatory effects including interferon induction (Kayser et al., 2001; Trun et al., 2006; Abdel Bar et al., 2022). Other ingredients that have been associated with pharmacological activities include catechin (antibacterial), scopoletin, epigallocatechin, taxifolin, and the putative EPs 7630 constituent epigallocatechin gallate (all antiviral) (Abdel Bar et al., 2022; Alossaimi et al., 2022; Emanuel et al., 2023).

Notably, the combination of different activities exerted by different constituents may contribute to the combined effects of *Pelargonium sidoides* extracts. For example, lower molecular weight sub-fractions of EPs 7630 displayed the most pronounced SARS-CoV-2 cell entry inhibition, but larger molecular weight fractions were most effective at inhibiting virus replication (Papies et al., 2021). Moreover, EPs 7630 fractions with limited antiviral activity displayed the most pronounced immunomodulatory activity in SARS-CoV-2-infected cells (Papies et al., 2021; Emanuel et al., 2023).

The situation is further complicated by the complex pharmacological and pharmacokinetic interactions of the constituents of plant extracts that can be difficult to impossible to decipher (McPartland and Pruitt, 1999; Wong et al., 2010; Yang et al., 2014; Wink, 2022; Skowrońska and Bazylko, 2023; Bailly, 2024). Hence, future research will have to (try to) disentangle further which ingredients are responsible for which pharmacological activities and to which extent EPs 7630 and other *Pelargonium sidoides* extracts have to be regarded as the active pharmaceutical ingredient, whose pharmacological activity depends on the combined effects of the large number of constituents and their multiple mechanisms of action.

The exact mechanisms of action of EPs 7630 are probably dependent on the disease types and may even differ between individuals suffering from the same illness. However, it seems obvious that the broad spectrum of clinical activities of EPs 7630 directly depends on its multiple pharmacological activities.

In particular, the combination of antiviral and immunomodulatory effects may enable EPs 7630 to tackle viral respiratory illnesses at different time points during the disease process. For example, the initial phases of COVID-19 are driven by SARS-CoV-2 replication (Brendler et al., 2021; Flerlage et al., 2021; Wong and Perlman, 2022), which is likely to be targeted by EPs 7630 via antiviral effects, including interferon-like activities. However, the later disease stages are caused by an overshooting immune response at a time point when the virus has already been cleared or when viral replication is very low (Brendler et al., 2021; Flerlage et al., 2021; Wong and Perlman, 2022). Based on the available data, it is plausible that EPs 7630 may also interfere with these late disease stages via its immunomodulatory effects. Moreover, the ability of EPs 7630 to induce an interferon response may be of particular interest for the prevention and treatment of COVID-19. Interferon inducers were recently suggested as desirable therapeutic options for the early treatment of SARS-CoV-2-infected patients at risk for severe COVID-19 (Savan and Gale, 2023).

Notably, diseases caused by many other respiratory viruses including influenza viruses and many common cold viruses are similarly driven by virus-induced and immunopathology-related symptoms (Newton et al., 2016; Flerlage et al., 2021; Wong and Perlman, 2022). Hence, all documented pharmacological mechanisms of EPs 7630 are probably essential for its broad-spectrum activity, and it may be difficult or even impossible to disentangle which mechanisms are responsible for the therapeutic effects observed in an individual case.

Finally, EPs 7630 is a prime example of a plant extract with evidence-based clinical efficacy that is approved for a range of indications in multiple countries (Conrad et al., 2007b; Malek and Funk, 2024). Its clinical activity is supported by a general understanding of the underlying mechanisms of action. This shows that plant extracts have a potential role as evidence-based treatments and deserve pre-clinical and clinical testing and investigation in the same way as any other drug class.
