# Supplementary material for: Multiple mechanisms enable broad-spectrum activity of the Pelargonium sidoides root extract EPs 7630 against acute respiratory tract infections
Source: Front Pharmacol. 2024 Oct 14;15:1455870. doi: 10.3389/fphar.2024.1455870 (PMC11513585; doi:10.3389/fphar.2024.1455870)
Supplement: Supplementary file 1 [file DataSheet2.PDF]

Suppl. Table 2. Effective antiviral concentrations of EPs 7630, other *Pelargonium sidoides* root extracts, and their ingredients.

**Assay**	**Extract (ingredient)**	**Virus**	**Activity**	**Reference**
Plaque reduction assay	*Pelargonium sidoides* crude aqueous extract	Herpes simplex virus type 1 strain KOS (in RC-37 cells)	0.00006% IC50*	Schnitzler et al., 2008
	Pelargonium sidoides crude aqueous extract	Herpes simplex virus type 2 strain HG52 (in RC-37 cells)	0.000005% IC50	Schnitzler et al., 2008
Cytopathogenic effect reduction assay	EPs 7630	Influenza A virus A/New Caledonia/20/99 (H1N1) (in MDCK cells)	9.45 µg/mL IC50	Michaelis et al., 2011
	EPs 7630	Influenza A virus A/California/7/2004 (H3N2) (in MDCK cells)	8.66 µg/mL IC50	Michaelis et al., 2011
	EPs 7630	Influenza A virus A/Thailand/1(Kan-1)/04 (H5N1) (in Vero cells)	>100 µg/mL IC50	Michaelis et al., 2011
	EPs 7630	Human coronavirus strain 229E (HCo-229E) (in Caco-2 cells)	44.5 µg/mL IC50	Michaelis et al., 2011
	EPs 7630	Coxsackie virus A9 (patient isolate) (in human foreskin fibroblasts)	14.8 µg/mL IC50	Michaelis et al., 2011
	EPs 7630	Respiratory syncytial virus (RSV) strain A2 ATCC VR-1540 (in Vero cells)	19.7 µg/mL IC50	Michaelis et al., 2011
	EPs 7630	Adenovirus 3 strain GB ATCC VR-3 (in Caco-2 cells)	>100 µg/mL IC50	Michaelis et al., 2011
	EPs 7630	Adenovirus 7 strain Gomen VR-7 (in Caco-2 cells)	>100 µg/mL IC50	Michaelis et al., 2011
	EPs 7630	Parainfluenza virus type 3 strain C243 ATCC VR-93 (in LLC-MK2 cells)	74.4 µg/mL IC50	Michaelis et al., 2011
	EPs 7630	human rhinovirus 16 strain 11757 ATCC VR-283 (in MelHO cells)	>100 µg/mL IC50	Michaelis et al., 2011
GFP fluorescence (using a GFP-expressing virus)	EPs 7630	Influenza A virus A/Puerto Rico/8/34-NS116-GFP (H1N1) (in A549 cells)	6.6 µg/mL	Theisen and Muller, 2012

Virus titre reduction	EPs 7630	Influenza A virus A/Luxembourg/46/2009 (H1N1/pdm09) (in A549 cells)	5.4 µg/mL IC50	Theisen and Muller, 2012
	EPs 7630	Adenovirus type 5 ATCC reference strain VR-1516 (in A549 cells)	>300 µg/mL IC50	Theisen and Muller, 2012
	EPs 7630	Measles virus Schwarz vaccine strain (in A549SLAM cells)	>300 µg/mL IC50	Theisen and Muller, 2012
GFP fluorescence (using a GFP-expressing virus)	Epigallocatechin	Influenza A virus A/Puerto Rico/8/34-NS116-GFP (H1N1) (in A549 cells)	42.5 µg/mL (139 µM) IC50	Theisen and Muller, 2012
	Gallocatechin	Influenza A virus A/Puerto Rico/8/34-NS116-GFP (H1N1) (in A549 cells)	28.4 µg/mL (92.7 µM) IC50	Theisen and Muller, 2012
	Epigallocatechin-(4β→8)-gallocatechin	Influenza A virus A/Puerto Rico/8/34-NS116-GFP (H1N1) (in A549 cells)	6.3 µg/mL (10.3 µM) IC50	Theisen and Muller, 2012
	Epigallocatechin-(4α→8)-epigallocatechin	Influenza A virus A/Puerto Rico/8/34-NS116-GFP (H1N1) (in A549 cells)	14.2 µg/mL (23.2 µM) IC50	Theisen and Muller, 2012
	Gallocatechin-(4β→8)-gallocatechin	Influenza A virus A/Puerto Rico/8/34-NS116-GFP (H1N1) (in A549 cells)	7.3 µg/mL (11.9 µM) IC50	Theisen and Muller, 2012
	Gallocatechin-(4α→8)-epigallocatechin	Influenza A virus A/Puerto Rico/8/34-NS116-GFP (H1N1) (in A549 cells)	13.0 µg/mL (21.2 µM) IC50	Theisen and Muller, 2012
	Oligo-/polymeric (epi-) gallocatechin EPs7630 fraction	Influenza A virus A/Puerto Rico/8/34-NS116-GFP (H1N1) (in A549 cells)	2.8 µg/mL IC50	Theisen and Muller, 2012
LC5-RIC cell fluorescent reporter signal assay	*Pelargonium sidoides* crude aqueous root extract	Clinical HIV isolate P-891	5.13 µg/mL IC50	Helfer et al., 2014
	Pelargonium sidoides crude aqueous root extract	Clinical HIV isolate CH077	3.95 µg/mL IC50	Helfer et al., 2014
	Pelargonium sidoides crude aqueous root extract	Clinical HIV isolate STCOr1	3.99 µg/mL	Helfer et al., 2014
	Pelargonium sidoides-derived polyphenols	Clinical HIV isolate P-891	6.67 µg/mL IC50	Helfer et al., 2014

	Pelargonium sidoides-derived polyphenols	Clinical HIV isolate CH077	4.00 µg/mL IC50	Helfer et al., 2014
	Pelargonium sidoides-derived polyphenols	HIV-1_{LAI}	1.00 µg/mL IC50	Helfer et al., 2014
Virus titre reduction	*Pelargonium sidoides* crude aqueous root extract	HIV-1_{LAI} (in LC5-RIC indicator cells), experiment 1	8.13 µg/mL IC50	Helfer et al., 2014
	Pelargonium sidoides crude aqueous root extract	HIV-1_{LAI} (in LC5-RIC indicator cells), experiment 2	8.00 µg/mL IC50	Helfer et al., 2014
	Pelargonium sidoides crude aqueous root extract	HIV-1_{LAI} (in human peripheral blood mononuclear cells, PBMCs)	5.70 µg/mL IC50	Helfer et al., 2014
	Pelargonium sidoides crude aqueous root extract	HIV-1_{LAI} (human monocyte-derived macrophages)	8.27 µg/mL IC50	Helfer et al., 2014
	Pelargonium sidoides-derived polyphenols	HIV-1_{LAI} (in human peripheral blood mononuclear cells, PBMCs)	2.98 µg/mL IC50	Helfer et al., 2014
Detection of rhinovirus-positive cells	EPs 7630	Rhinovirus 16 clinical isolate (in primary human bronchial epithelial cells)	Significant reduction of rhinovirus-positive cells by 10µg/mL (numbers not provided)	Roth et al., 2019
Cytopathogenic effect reduction assay	*Pelargonium sidoides* root extract	Rhinovirus A2 (in HeLa Ohio cells)	>100 µg/mL IC50	Walther et al., 2020
	Pelargonium sidoides root extract	Rhinovirus B14 (in HeLa Ohio cells)	>100 µg/mL IC50	Walther et al., 2020
	Pelargonium sidoides root extract	Influenza A virus A/Hong Kong/68 (H3N2) (in HeLa Ohio cells)	11.67 µg/mL IC50	Walther et al., 2020
	Pelargonium sidoides root extract	Influenza A virus A/Jena/8178/09 (H1N1pdm09) (in HeLa Ohio cells)	7.80 µg/mL IC50	Walther et al., 2020
	Pelargonium sidoides root extract	Influenza A virus A/Hong Kong/68 (H3N2) (in MDCK cells)	~10 µg/mL IC50	Walther et al., 2020
	Pelargonium sidoides root extract	Influenza A virus A/Jena/8178/09	~10 µg/mL IC50	Walther et al., 2020

		(H1N1pdm09) (in MDCK cells)		
Plaque titration assay	EPs 7630	SARS-CoV-2 strain Munich/2020/984 (in Calu-3 cells)	1.61 µg/mL IC50	Papies et al., 2021
		SARS-CoV-2 strain Munich/2020/984 (in VeroFM cells)	0.48 µg/mL IC50	Papies et al., 2021
		Mumps virus (in Calu-3 cells)	>100 µg/ mL IC50	Papies et al., 2021
Cytopathogenic effect reduction assay	EPs 7630	SARS-CoV-2 strain hCoV-19/Egypt/NRC-03/2020 (in Vero E6 cells)	13.79 µg/mL IC50	Alossaimi et al., 2022
	Scopoletin	SARS-CoV-2 strain hCoV-19/Egypt/NRC-03/2020 (in Vero E6 cells)	17.79 µM IC50	Alossaimi et al., 2022

[1] IC50 = 50% inhibitory concentration
