# Supplementary material for: Multiple mechanisms enable broad-spectrum activity of the Pelargonium sidoides root extract EPs 7630 against acute respiratory tract infections
Source: Front Pharmacol. 2024 Oct 14;15:1455870. doi: 10.3389/fphar.2024.1455870 (PMC11513585; doi:10.3389/fphar.2024.1455870)
Supplement: Supplementary file 2 [file DataSheet1.PDF]

Suppl. Table 1. Effective antibacterial concentrations of EPs 7630, other *Pelargonium sidoides* root extracts, and their ingredients.

Assay	Extract (ingredient)	Bacterial strain	Activity	Reference
Agar dilution method	*Pelargonium sidoides* crude root extract	*Escherichia coli* ATCC 25922	5 mg/mL MIC[1]	Kayser and Kolodziej, 1997
	Pelargonium sidoides crude root extract	*Klebsiella pneumoniae* V 6089	7.5 mg/mL MIC	Kayser and Kolodziej, 1997
	Pelargonium sidoides crude root extract	*Proteus mirabilis* ATCC 14153	5 mg/mL MIC	Kayser and Kolodziej, 1997
	Pelargonium sidoides crude root extract	*Pseudomonas aeruginosa* ATCC 27853	5 mg/mL MIC	Kayser and Kolodziej, 1997
	Pelargonium sidoides crude root extract	*Haemophilus influenzae* ATCC 33379	5 mg/mL MIC	Kayser and Kolodziej, 1997
	Pelargonium sidoides crude root extract	*Staphylococcus aureus* ATCC 25923	7.5 mg/mL MIC	Kayser and Kolodziej, 1997
	Pelargonium sidoides crude root extract	Beta-hemolytic *Streptococcus* 1451	7.5 mg/mL MIC	Kayser and Kolodziej, 1997
	Pelargonium sidoides crude root extract	*Streptococcus pneumoniae* (strain 78)	7.5 mg/mL MIC	Kayser and Kolodziej, 1997
Agar dilution method	EPs 7630	*Klebsiella pneumoniae* V 6089	13.8 mg/mL MIC	Kolodziej et al., 2003
	EPs 7630	*Escherichia coli* ATCC 25922	>13.8 mg/mL MIC	Kolodziej et al., 2003
	EPs 7630	*Pseudomonas aeruginosa* ATCC 27853	>13.8 mg/mL MIC	Kolodziej et al., 2003
	EPs 7630	*Proteus mirabilis* ATCC 14153	3.3 mg/mL MIC	Kolodziej et al., 2003
	EPs 7630	*Staphylococcus aureus* ATCC 25923	3.3 mg/mL MIC	Kolodziej et al., 2003
	EPs 7630	*Staphylococcus aureus* 1150.92 (multi-resistant)	3.3 mg/mL MIC	Kolodziej et al., 2003
	EPs 7630	*Staphylococcus aureus* 1583.93 (multi-resistant)	3.3 mg/mL MIC	Kolodziej et al., 2003
	EPs 7630	*Staphylococcus aureus* 999.93 (multi-resistant)	3.3 mg/mL MIC	Kolodziej et al., 2003
	EPs 7630	*Staphylococcus aureus* 134.93 (multi-resistant)	3.3 mg/mL MIC	Kolodziej et al., 2003
	EPs 7630	*Staphylococcus aureus* 1000.93 (multi-resistant)	3.3 mg/mL MIC	Kolodziej et al., 2003

Radiorespirometric bioassay (BACTEC 460 system)	*Pelargonium sidoides* crude root extract	*Mycobacterium tuberculosis* strain H37Rv ATCC 27294	96% growth inhibition at 12.5 µg/mL	Kolodziej et al., 2003
Alamar blue assay	*Pelargonium sidoides* crude root extract	*Mycobacterium tuberculosis* strain H37Rv ATCC 27294	100 µg/mL MIC (control: rifampicin 0.06 µg/mL MIC)	Kolodziej et al., 2003
Microdilution assay	Oleic acid	*Mycobacterium aurum* A+	4 mg/mL MIC (controls: streptomycin 0.5 mg/mL MIC, isoniazid 0.06 mg/mL MIC)	Seidel and Taylor, 2004
	Oleic acid	*Mycobacterium smegmatis* ATCC 14468	128 mg/mL MIC (controls: streptomycin 0.5 mg/mL MIC, isoniazid 1 mg/mL MIC)	Seidel and Taylor, 2004
	Oleic acid	*Mycobacterium fortuitum* ATCC 6841	≥256 mg/mL MIC (control: doxycycline ≤0.13 mg/mL MIC)	Seidel and Taylor, 2004
	Oleic acid	*Mycobacterium abscessus* ATCC 19977	≥256 mg/mL MIC (control: clarithromycine ≤0.13 mg/mL MIC)	Seidel and Taylor, 2004
	Oleic acid	*Mycobacterium phlei* ATCC 11758	16 mg/mL MIC	Seidel and Taylor, 2004
	Linoleic acid	*Mycobacterium aurum* A+	2 mg/mL MIC (controls: streptomycin 0.5 mg/mL MIC, isoniazid 0.06 mg/mL MIC)	Seidel and Taylor, 2004
	Linoleic acid	*Mycobacterium smegmatis* ATCC 14468	4 mg/mL MIC (controls: streptomycin 0.5 mg/mL MIC, isoniazid 1 mg/mL MIC)	Seidel and Taylor, 2004
	Linoleic acid	*Mycobacterium fortuitum* ATCC 6841	16 mg/mL MIC (control: doxycycline ≤0.13 mg/mL MIC)	Seidel and Taylor, 2004
	Linoleic acid	*Mycobacterium abscessus* ATCC 19977	16-32 mg/mL MIC (control: clarithromycine	Seidel and Taylor, 2004

			≤0.13 mg/mL MIC)	
	Linoleic acid	*Mycobacterium phlei* ATCC 11758	2-4 mg/mL MIC	Seidel and Taylor, 2004
Microdilution assay	*Pelargonium sidoides* buthanol root extract	*Mycobacterium smegmatis* MC^2 155	0.156 µg/mL MIC (control: ciprofloxacin 0.125 µg/mL MIC)	Mativandlela et al., 2007
	Scopoletin	*Mycobacterium smegmatis* MC^2 155	7.81 µg/mL MIC (control: ciprofloxacin 0.125 µg/mL MIC)	Mativandlela et al., 2007
	Umckalin	*Mycobacterium smegmatis* MC^2 155	62.5 µg/mL MIC (control: ciprofloxacin 0.125 µg/mL MIC)	Mativandlela et al., 2007
	Catechin	*Mycobacterium smegmatis* MC^2 155	31.25 µg/mL MIC (control: ciprofloxacin 0.125 µg/mL MIC)	Mativandlela et al., 2007
	Epigallocatechin	*Mycobacterium smegmatis* MC^2 155	7.81 µg/mL MIC (control: ciprofloxacin 0.125 µg/mL MIC)	Mativandlela et al., 2007
Radiorespirometric bioassay (BACTEC 460 system)	*Pelargonium sidoides* buthanol root extract	*Mycobacterium tuberculosis* strain H37Rv ATCC 27294	2.5 mg/mL MIC	Mativandlela et al., 2007
	Scopoletin	*Mycobacterium tuberculosis* strain H37Rv ATCC 27294	>200 µg/mL MIC	Mativandlela et al., 2007
	Umckalin	*Mycobacterium tuberculosis* strain H37Rv ATCC 27294	>200 µg/mL MIC	Mativandlela et al., 2007
	Catechin	*Mycobacterium tuberculosis* strain H37Rv ATCC 27294	>200 µg/mL MIC	Mativandlela et al., 2007
	Epigallocatechin	*Mycobacterium tuberculosis* strain H37Rv ATCC 27294	>200 µg/mL MIC	Mativandlela et al., 2007
Turbidity (600 nm) measurement	EPs 7630	*Helicobacter pylori*	43% growth reduction by 100 µg/mL	Beil and Kilian, 2007
Disk diffusion test	EPs 7630	*Helicobacter pylori*	No growth inhibition at 10 mg/mL	Wittschier et al., 2007a
Alamar blue assay	*Pelargonium sidoides* crude root extract	*Streptococcus salivarius* DSM 20067	86.3% viability reduction by 0.09 g/mL	Savickiene et al., 2018

	Pelargonium sidoides crude root extract	*Porphyromonas gingivalis* DSM 20709	90.3% viability reduction by 0.09 g/mL	Savickiene et al., 2018
	Pelargonium sidoides proanthocyanidin fraction	*Streptococcus salivarius* DSM 20067	57.0% viability reduction by 0.09 g/mL	Savickiene et al., 2018
	Pelargonium sidoides proanthocyanidin fraction	*Porphyromonas gingivalis* DSM 20709	91.5% viability reduction by 0.09 g/mL	Savickiene et al., 2018
Alamar blue assay	*Pelargonium sidoides* crude root extract	*Staphylococcus aureus* ATCC43300	75% viability reduction by 80µg/ mL	Jekabsone et al., 2019
	Pelargonium sidoides crude root extract	*Aggregatibacter actinomycetemcomitans* ATCC33384	57% viability reduction by 80µg/ mL	Jekabsone et al., 2019
	Pelargonium sidoides crude root extract	*Staphylococcus epidermidis* clinical isolate	97% viability reduction by 80µg/ mL	Jekabsone et al., 2019
	Pelargonium sidoides crude root extract	*Escherichia coli* (non-pathogenic) ATCC BAA-1427	26% viability reduction by 80µg/ mL	Jekabsone et al., 2019
	Pelargonium sidoides proanthocyanidin fraction	*Staphylococcus aureus* ATCC43300	47% viability reduction by 80µg/ mL	Jekabsone et al., 2019
	Pelargonium sidoides proanthocyanidin fraction	*Aggregatibacter actinomycetemcomitans* ATCC33384	99% viability reduction by 80µg/ mL	Jekabsone et al., 2019
	Pelargonium sidoides proanthocyanidin fraction	*Staphylococcus epidermidis* clinical isolate	96% viability reduction by 80µg/ mL	Jekabsone et al., 2019
	Pelargonium sidoides proanthocyanidin fraction	*Escherichia coli* (non-pathogenic) ATCC BAA-1427	47% viability reduction by 80µg/ mL	Jekabsone et al., 2019
Agar well diffusion method	Catechin	*Pseudomonas aeruginosa* ATCC 27853	16 µg/mL MIC (control: ciprofloxacin 0.5 µg/mL MIC)	Abdel Bar et al., 2022
	Catechin	*Pseudomonas aeruginosa* clinical isolate (P1)	64 µg/mL MIC (control: ciprofloxacin 16 µg/mL MIC)	Abdel Bar et al., 2022
	Catechin	*Pseudomonas aeruginosa* clinical isolate (P2)	1024 µg/mL MIC (control: ciprofloxacin	Abdel Bar et al., 2022

			32 µg/mL MIC)	
	Catechin	*Pseudomonas aeruginosa* clinical isolate (P3)	1024 µg/mL MIC (control: ciprofloxacin 32 µg/mL MIC)	Abdel Bar et al., 2022
	Catechin	*Pseudomonas aeruginosa* clinical isolate (P4)	64 µg/mL MIC (control: ciprofloxacin 16 µg/mL MIC)	Abdel Bar et al., 2022
	Catechin	*Pseudomonas aeruginosa* clinical isolate (P5)	256 µg/mL MIC (control: ciprofloxacin 32 µg/mL MIC)	Abdel Bar et al., 2022
	Catechin	*Pseudomonas aeruginosa* clinical isolate (P6)	512 µg/mL MIC (control: ciprofloxacin 32 µg/mL MIC)	Abdel Bar et al., 2022
	Catechin	*Pseudomonas aeruginosa* clinical isolate (P7)	128 µg/mL MIC (control: ciprofloxacin 32 µg/mL MIC)	Abdel Bar et al., 2022
	Catechin	*Pseudomonas aeruginosa* clinical isolate (P8)	64 µg/mL MIC (control: ciprofloxacin 16 µg/mL MIC)	Abdel Bar et al., 2022
	Catechin	*Pseudomonas aeruginosa* clinical isolate (P9)	1024 µg/mL MIC (control: ciprofloxacin 16 µg/mL MIC)	Abdel Bar et al., 2022
	Catechin	*Pseudomonas aeruginosa* clinical isolate (P10)	512 µg/mL MIC (control: ciprofloxacin 256 µg/mL MIC)	Abdel Bar et al., 2022
	Catechin	*Pseudomonas aeruginosa* clinical isolate (P11)	64 µg/mL MIC (control: ciprofloxacin 128 µg/mL MIC)	Abdel Bar et al., 2022
	Catechin	*Pseudomonas aeruginosa* clinical isolate (P12)	1024 µg/mL MIC (control: ciprofloxacin 16 µg/mL MIC)	Abdel Bar et al., 2022

	Catechin	*Pseudomonas aeruginosa* clinical isolate (P13)	512 µg/mL MIC (control: ciprofloxacin 16 µg/mL MIC)	Abdel Bar et al., 2022
	Catechin	*Pseudomonas aeruginosa* clinical isolate (P14)	128 µg/mL MIC (control: ciprofloxacin 32 µg/mL MIC)	Abdel Bar et al., 2022
	Catechin	*Pseudomonas aeruginosa* clinical isolate (P15)	512 µg/mL MIC (control: ciprofloxacin 32 µg/mL MIC)	Abdel Bar et al., 2022
	Catechin	*Pseudomonas aeruginosa* clinical isolate (P16)	64 µg/mL MIC (control: ciprofloxacin 16 µg/mL MIC)	Abdel Bar et al., 2022
	Catechin	*Pseudomonas aeruginosa* clinical isolate (P17)	512 µg/mL MIC (control: ciprofloxacin 32 µg/mL MIC)	Abdel Bar et al., 2022
	Catechin	*Pseudomonas aeruginosa* clinical isolate (P18)	512 µg/mL MIC (control: ciprofloxacin 256 µg/mL MIC)	Abdel Bar et al., 2022
	Catechin	*Pseudomonas aeruginosa* clinical isolate (P19)	16 µg/mL MIC (control: ciprofloxacin 0.5 µg/mL MIC)	Abdel Bar et al., 2022
	Gallic acid	*Pseudomonas aeruginosa* ATCC 27853	32 µg/mL MIC (control: ciprofloxacin 0.5 µg/mL MIC)	Abdel Bar et al., 2022
	Gallic acid	*Pseudomonas aeruginosa* clinical isolate (P1)	128 µg/mL MIC (control: ciprofloxacin 16 µg/mL MIC)	Abdel Bar et al., 2022
	Gallic acid	*Pseudomonas aeruginosa* clinical isolate (P2)	512 µg/mL MIC (control: ciprofloxacin 32 µg/mL MIC)	Abdel Bar et al., 2022
	Gallic acid	*Pseudomonas aeruginosa* clinical isolate (P3)	128 µg/mL MIC (control: ciprofloxacin	Abdel Bar et al., 2022

			32 µg/mL MIC)	
	Gallic acid	*Pseudomonas aeruginosa* clinical isolate (P4)	256 µg/mL MIC (control: ciprofloxacin 16 µg/mL MIC)	Abdel Bar et al., 2022
	Gallic acid	*Pseudomonas aeruginosa* clinical isolate (P5)	128 µg/mL MIC (control: ciprofloxacin 32 µg/mL MIC)	Abdel Bar et al., 2022
	Gallic acid	*Pseudomonas aeruginosa* clinical isolate (P6)	1024 µg/mL MIC (control: ciprofloxacin 32 µg/mL MIC)	Abdel Bar et al., 2022
	Gallic acid	*Pseudomonas aeruginosa* clinical isolate (P7)	512 µg/mL MIC (control: ciprofloxacin 32 µg/mL MIC)	Abdel Bar et al., 2022
	Gallic acid	*Pseudomonas aeruginosa* clinical isolate (P8)	512 µg/mL MIC (control: ciprofloxacin 16 µg/mL MIC)	Abdel Bar et al., 2022
	Gallic acid	*Pseudomonas aeruginosa* clinical isolate (P9)	1024 µg/mL MIC (control: ciprofloxacin 16 µg/mL MIC)	Abdel Bar et al., 2022
	Gallic acid	*Pseudomonas aeruginosa* clinical isolate (P10)	128 µg/mL MIC (control: ciprofloxacin 256 µg/mL MIC)	Abdel Bar et al., 2022
	Gallic acid	*Pseudomonas aeruginosa* clinical isolate (P11)	128 µg/mL MIC (control: ciprofloxacin 128 µg/mL MIC)	Abdel Bar et al., 2022
	Gallic acid	*Pseudomonas aeruginosa* clinical isolate (P12)	512 µg/mL MIC (control: ciprofloxacin 16 µg/mL MIC)	Abdel Bar et al., 2022
	Gallic acid	*Pseudomonas aeruginosa* clinical isolate (P13)	64 µg/mL MIC (control: ciprofloxacin 16 µg/mL MIC)	Abdel Bar et al., 2022

	Gallic acid	*Pseudomonas aeruginosa* clinical isolate (P14)	128 µg/mL MIC (control: ciprofloxacin 32 µg/mL MIC)	Abdel Bar et al., 2022
	Gallic acid	*Pseudomonas aeruginosa* clinical isolate (P15)	128 µg/mL MIC (control: ciprofloxacin 32 µg/mL MIC)	Abdel Bar et al., 2022
	Gallic acid	*Pseudomonas aeruginosa* clinical isolate (P16)	1024 µg/mL MIC (control: ciprofloxacin 16 µg/mL MIC)	Abdel Bar et al., 2022
	Gallic acid	*Pseudomonas aeruginosa* clinical isolate (P17)	256 µg/mL MIC (control: ciprofloxacin 32 µg/mL MIC)	Abdel Bar et al., 2022
	Gallic acid	*Pseudomonas aeruginosa* clinical isolate (P18)	1024 µg/mL MIC (control: ciprofloxacin 256 µg/mL MIC)	Abdel Bar et al., 2022
	Gallic acid	*Pseudomonas aeruginosa* clinical isolate (P19)	256 µg/mL MIC (control: ciprofloxacin 0.5 µg/mL MIC)	Abdel Bar et al., 2022

[1] MIC = Minimal inhibitory concentration
